# Supplementary material for: Combined Transcriptomics and Metabolomics Analysis Reveals the Effect of Selenium Fertilization on Lycium barbarum Fruit
Source: Molecules. 2023 Dec 14;28(24):8088. doi: 10.3390/molecules28248088 (PMC10745541; doi:10.3390/molecules28248088)
Supplement: Supplementary file 1 [file molecules-28-08088-s001.zip › Figure S1.pdf]

A

SE1vsCK

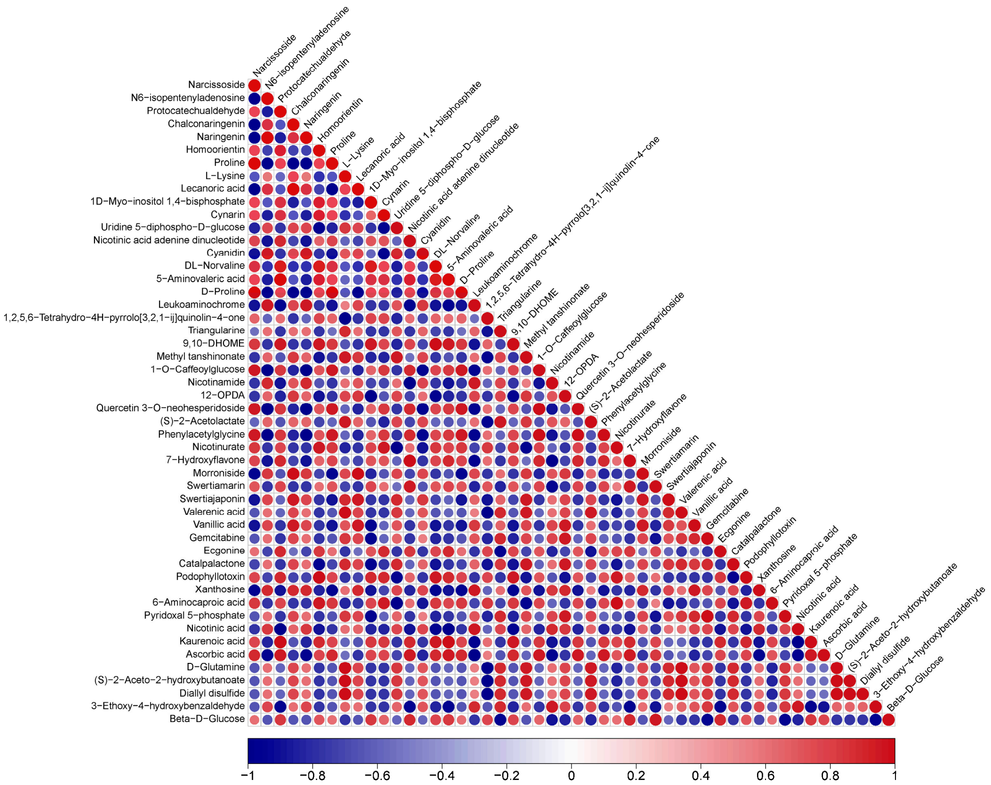

Narcissoside
N6-isopentenyladenosine
Protocatechualdehyde
Chalconaringenin
Naringenin
Homoorientin
Proline
L-Lysine
Lecanoric acid
1D-Myo-inositol 1,4-bisphosphate
Cynarin
Uridine 5-diphospho-D-glucose
Nicotinic acid adenine dinucleotide
Cyanidin
DL-Norvaline
5-Aminovaleric acid
D-Proline
Leukoaminochrome
1,2,5,6-Tetrahydro-4H-pyrrolo[3,2,1-ij]quinolin-4-one
Triangularine
9,10-DHOME
Methyl tanshinonate
1-O-Caffeoylglucose
Nicotinamide
12-OPDA
Quercetin 3-O-neohesperidoside
(S)-2-Acetolactate
Phenylacetylglycine
Nicotinurate
7-Hydroxyflavone
Morroniside
Swertiamarin
Swertiajaponin
Valerenic acid
Vanillic acid
Gemcitabine
Ecgonine
Catalpalactone
Podophyllotoxin
Xanthosine
6-Aminocaproic acid
Pyridoxal 5-phosphate
Nicotinic acid
Kaurenoic acid
Ascorbic acid
D-Glutamine
(S)-2-Aceto-2-hydroxybutanoate
Diallyl disulfide
3-Ethoxy-4-hydroxybenzaldehyde
Beta-D-Glucose
-1
-0.8
-0.6
-0.4
-0.2
0
0.2
0.4
0.6
0.8
1

B

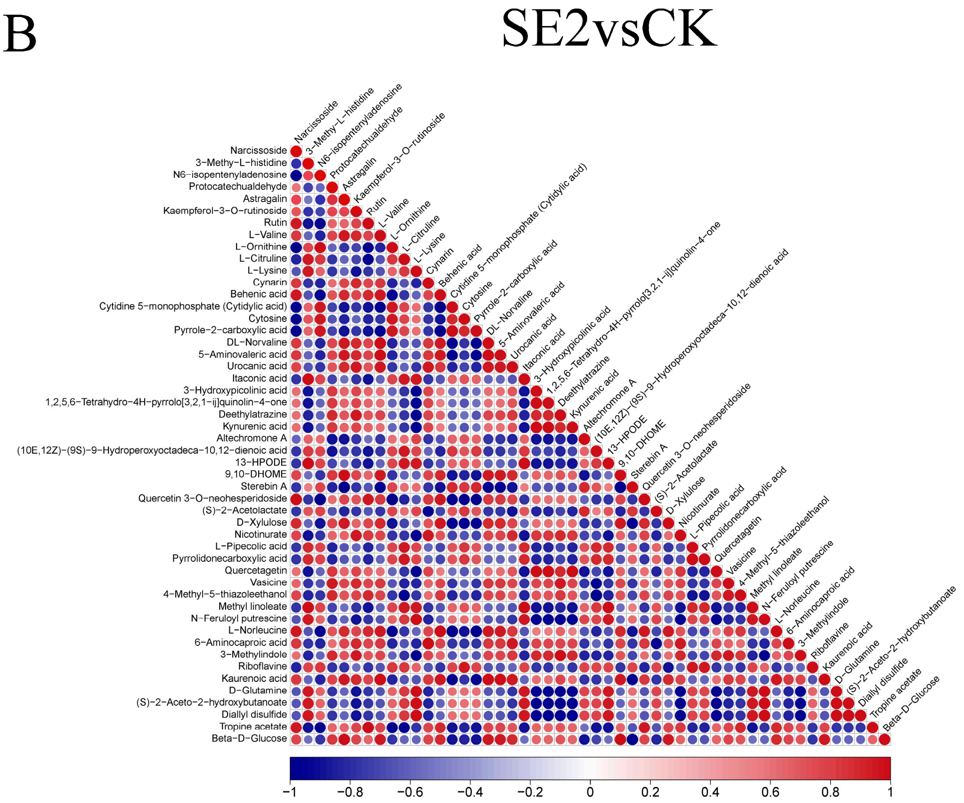
SE2vsCK
Narcissoside
3−Methy−L−histidine
N6−isopentenyladenosine
Protocatechualdehyde
Astragalin
Kaempferol−3−O−rutinoside
Rutin
L−Valine
L−Ornithine
L−Citruline
L−Lysine
Cynarin
Behenic acid
Cytidine 5−monophosphate (Cytidylic acid)
Cytosine
Pyrrole−2−carboxylic acid
DL−Norvaline
5−Aminovaleric acid
Urocanic acid
Itaconic acid
3−Hydroxypicolinic acid
1,2,5,6−Tetrahydro−4H−pyrrolo[3,2,1−ij]quinolin−4−one
Deethylatrazine
Kynurenic acid
Altechromone A
(10E,12Z)−(9S)−9−Hydroperoxyoctadeca−10,12−dienoic acid
13−HPODE
9,10−DHOME
Sterebin A
Quercetin 3−O−neohesperidoside
(S)−2−Acetolactate
D−Xylulose
Nicotinurate
L−Pipecolic acid
Pyrrolidonecarboxylic acid
Quercetagetin
Vasicine
4−Methyl−5−thiazoleethanol
Methyl linoleate
N−Feruloyl putrescine
L−Norleucine
6−Aminocaproic acid
3−Methylindole
Riboflavine
Kaurenoic acid
D−Glutamine
(S)−2−Aceto−2−hydroxybutanoate
Diallyl disulfide
Tropine acetate
Beta−D−Glucose
−1
−0.8
−0.6
−0.4
−0.2
0
0.2
0.4
0.6
0.8
1

C

SE3vsCK

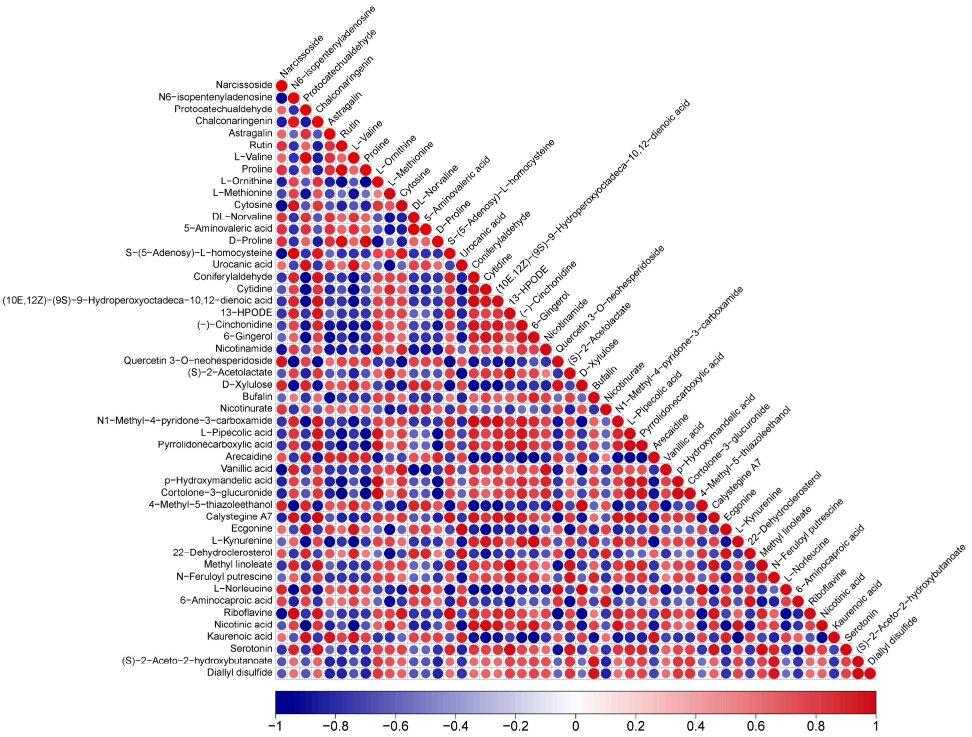

Figure S1. Correlation analysis of differential metabolites in *Lycium barbarum* samples ((A)-Sodium selenite (SE1), (B)-Nano-selenium (SE2), (C)-Organic selenium(SE3), versus sprayed water control (CK)).
