# Supplementary material for: Combined Transcriptomics and Metabolomics Analysis Reveals the Effect of Selenium Fertilization on Lycium barbarum Fruit
Source: Molecules. 2023 Dec 14;28(24):8088. doi: 10.3390/molecules28248088 (PMC10745541; doi:10.3390/molecules28248088)
Supplement: Supplementary file 1 [file molecules-28-08088-s001.zip › Supplementary Data.pdf]

Supplementary Data

Supplementary Figure

Figure S1. Correlation analysis of differential metabolites in *Lycium barbarum* samples ((A)-Sodium selenite (SE1), (B)-Nano-selenium (SE2), (C)-Organic selenium(SE3), versus sprayed water control (CK))

Table S1. Differential metabolites related to amino acid metabolism in *Lycium barbarum*

Table S2. Differential metabolites related to flavonoids and flavonoid metabolism in *Lycium barbarum*

TableS3. Differential metabolites related to the alkaloid metabolism in *Lycium barbarum*

Note: The list of standard compounds used for MRM analysis
